# Cases of Ischuria Renalis in Children

**Published:** 1792

**Authors:** Robert Willan

**Affiliations:** Physician to the Public Dispensary in London.


### MEDICAL FACTS

AND

### OBSERVATIONS.

I. *Cases of Ischuria Renalis in Children. By* Robert Willan, *M. D. F. A. S. Physician to the Public Dispensary in London.*

IN the course of the year 1784 I observed three instances of sudden death in children who had not been previously affected with any violent or alarming complaint.

The train of symptoms was nearly as follows: —At first a slight feverish heat, restlessness, diarrhœa, and sometimes bilious vomiting, which continued for about a week; during that time the urine was made in small quantity, till at length the discharge of it entirely ceased, and soon afterward the patients died unexpectedly,

 without without complaining of pain or any particular uneafinefs.

No medical affiftance having been thought requifite in the firft days of the difeafe, I did not, therefore, fee any of thefe cafes till the fuppreffion of urine had taken place, and at that time found the diarrhœa abated, the pulfe and ftate of the fkin natural.

The predominant fymptom, ifchuria, was the only one which demanded immediate attention. As there appeared no painful tenfion or fwelling in the hypogaftric region, nor in any part of the abdomen, I had hopes of affording fpeedy relief by means of cooling diuretics, clyfters, and fomentations.

Thefe applications, however, and other remedies which the circumftances fuggefted, were infufficient to reftore the difcharge of urine before the patient fell a victim to the difeafe.

To be thus difappointed in fo many cafes, occurring nearly together, made me very uneafy. I found no fatisfactory information in medical authors, nor from practitioners with whom I converfed on the fubject: I determined, however, if a fimilar cafe fhould again occur, to employ diligently the femicupium, or warm bath, as a remedy moſt generally efficacious in the iſchuria renalis.

Two years afterwards an opportunity occurred of adopting this plan in the caſe of Maſter K., a fine ſenſible boy about nine years old. In the beginning of October, 1786, he had the ſcarlatina anginoſa, of which he ſoon recovered under the care of Mr. Thomas Auſtin, his apothecary. He continued perfectly well for about a week, went out every day, and amuſed himſelf as uſual. It was then obſerved that he began to loſe his appetite, and to be ſomewhat debilitated: he had alſo frequent, though ſlight, attacks of ſickneſs and diarrhœa.

On the 23d and 24th of October he ſeemed much better; but on the 25th found a total inability to make water.

Mr. Auſtin, to whom I had formerly communicated my obſervations reſpecting the inſidious nature of theſe complaints in young ſubjects, deſired me to ſee this patient early on the 26th. I found him then eaſy and compoſed: his ſkin was cool, his pulſe at 90, and he had no mark of fever, except a ſlight yellowiſh-brown fur on the back part of the tongue. His cheeks were rather bloated; but he had no pain of the head or ſtomach: his abdomen was not in any part tenſe or ſwelled, nor had he any ſenſe of pain on its being preſſed.

After preſcribing a gentle laxative, I recommended the warm bath as the remedy to be principally truſted.

He was kept in the bath, on the firſt application of it, twenty minutes. Afterwards, as he ſtood on the floor, he made a ſmall glaſsful of water. No internal miſchief could be detected from the ſtate of this urine, which was clear and of a natural colour.

In the evening he was briſk and lively: his cheeks did not appear ſo much bloated. I directed that the bath ſhould be again employed, hoping it might produce an effect as great as before, or even greater.

Next morning he was nearly in the ſame ſtate; but he had made only a few drops of water on coming out of the bath. I then deſired he might be kept in it three quarters of an hour, and attempt to make water in that ſituation, or afterwards ſtanding on the cold floor.

At night I was informed that theſe directions had been attended with little ſucceſs; and farther, that during the day he had been ſeized with with a kind of fit, in which, after a ſudden ſhivering, he became very cold and inſenſible, having his eyes fixed for ſome minutes. He ſoon, however, recovered from this ſtate, and when I ſaw him again was ſerene and cheerful as on the preceding day.

Though the ſymptoms were not aggravated ſince my firſt viſit, ſtill he was evidently in a very precarious ſituation. So much watery fluid could not be retained in the blood, the urinary diſcharge being ſuppreſſed, without inducing fatal conſequences from its effuſion on the brain, a circumſtance ſo frequent in ſimilar caſes.

A conſultation was propoſed, to which I could not but readily accede: this was, however, deferred till next day, it being then late in the evening. I deſired, in the mean time, that a fourth trial might be made of the warm bath.

At eleven o'clock he had ſtrength enough to get in and out of the bath without aſſiſtance. He then went to bed, and talked cheerfully to thoſe about him till twelve o'clock, when he ſuddenly complained that he could not ſee, and very ſoon after expired in a fit ſimilar to that with which he had been affected in the courſe of the day.

An examination of the diſeaſed parts not being permitted, I could not thence confirm or diſprove the ideas I had formed reſpecting the complaint from the preſent caſe.

Another inſtance of the ſame kind occurred March 20th, 1787, in a child about two years old. — His mother informed me, that ſix days before he had been taken with a diarrhœa and vomiting. Thoſe ſymptoms abated within a day or two, but a cough and ſome degree of fever ſtill remained. The diſcharge of urine had ceaſed upwards of twenty-four hours before he was brought to me: he was then very reſtleſs; his breath was ſhort; his pulſe ſmall and quick; his face was bloated, and of a ſallow or almoſt livid hue; but there appeared no hardneſs or ſwelling of the abdomen.

As the caſe was evidently in its extreme ſtate, I deemed it unneceſſary to direct the application of any remedies, and preſently learnt that the child died within an hour after its return home.

Being allowed to inſpect the viſcera of this patient, I at length diſcovered the ſtate of the parts affected in this dangerous malady. The whole of the meſentery appeared to have been inflamed.

inflamed. The inflammation had extended thence to a confiderable portion of the ileum, on which were alfo found two gangrenous fpots, each of them about the fize of a fixpence.

There was no urine in the bladder: the ftomach, liver, and kidnies, were in a natural ftate; the gall bladder was very turgid, and the mefenteric glands were much enlarged.

In the courfe of my attendance on the above patients I had been led to conclude that the fymptoms arofe from an inflammation of fome of the abdominal vifcera. The laft cafe, which confirmed that opinion, alfo limited the feat of inflammation to the mefentery. The circumftances appearing in all of them were fo fimilar, that they might reafonably be referred to the fame caufe, and probably the common termination was by gangrene; at leaft I am inclined to think this muft have happened to Mafter K., at an early period, even before my firft vifit to him, which is rendered more probable from his having had violent fhiverings, with coldnefs and infenfibility, for a confiderable time, on the 22d of October, when the complaint in his bowels firſt abated, a circumſtance of which I was not informed till afterwards.

It farther appears, from all theſe caſes, that meſenteric inflammation has the ſame ſlow and obſcure courſe of ſymptoms as inflammation affecting the omentum, peritonæum, and mediaſtinum.

The beſt account of meſenteritis is given by Riverius *. It coincides in moſt particulars with the caſes which I have related above; but neither Riverius nor any other author has mentioned the renal iſchuria as a ſymptom of the diſorder.

On comparing together the caſes above related, I was induced to think that the ſuppreſſion of urine only came on after gangrene had taken place; but another caſe which has ſince occurred proves the contrary.

* "Signa inflammationis meſenterii diagnoſtica ſunt "febris languida, occulta et lenta, abſque ſiti & gravioribus "ſymptomatis: anorexia; tenſionis & gravitatis ſenſus "ſubter ventriculum, citra magnam duritiem & quæ non "niſi preſſu dignoſcitur: citra dolorem etiam, ſaltem ef-"fatu dignum, quia pars obtuſo ſenſu donata eſt: ejec-"tiones chyloſæ quas plerumque ichor tenuis conſequitur "ſine ullo doloris ſenſu, modo ſeorſim & ſincerus, modo "fæcibus permixtus."—*Vide* Riverii Praxeos Med. Lib. xiii. cap. 2.

A child

A child, three years old, was brought to the Publick Difpenfary on the 28th of September, 1789. This patient had made no urine for upwards of eighteen hours. The prior fymptoms had been nearly fimilar to thofe mentioned in the foregoing accounts. He was uneafy and fretful, had a quick fmall pulfe, but no fur upon the tongue, nor any degree of tenfion in the abdomen.

I ordered eight leeches to be applied to the abdomen, and a blifter near the os facrum; and was informed on the following day that the urinary fecretion had been fully reftored. The difcharge continued afterwards in regular order, and the ftate of his bowels was natural. His original difeafe, therefore, appeared to be completely removed; but in the courfe of a few days he again became languid and heavy; and I am forry to add, that he died about a month after with fymptoms of hydrocephalus diftinctly marked.

As the above cafes all occurred in infants or young children, it might feem probable that ifchuria, as a fymptom of mefenteric inflammation, is peculiar to them; but this conclufion would not be juft, as I have feen one fatal cafe case of the same kind at adult age, attended with the usual insidious train of symptoms.

